# Allosteric coupling between proximal C-terminus and selectivity filter is facilitated by the movement of transmembrane segment 4 in TREK-2 channel

**DOI:** 10.1038/srep21248

**Published:** 2016-02-16

**Authors:** Ren-Gong Zhuo, Peng Peng, Xiao-Yan Liu, Hai-Tao Yan, Jiang-Ping Xu, Jian-Quan Zheng, Xiao-Li Wei, Xiao-Yun Ma

**Affiliations:** 1 State Key Laboratory of Toxicology and Medical Countermeasures, Beijing Key Laboratory of Neuropsychopharmacology, Department of Biochemical Pharmacology, Beijing Institute of Pharmacology and Toxicology, Beijing 100850, China; 2 Anesthesia and Operation Center, PLA General Hospital, Beijing 100853, China; 3 Department of Pharmacology, School of Pharmaceutical Sciences, Southern Medical University, Guangzhou 510515, China

## Abstract

TREK-2, a member of two-pore-domain potassium channel family, regulates cellular excitability in response to diverse stimuli. However, how such stimuli control channel function remains unclear. Here, by characterizing the responses of cytosolic proximal C-terminus deletant (ΔpCt) and transmembrane segment 4 (M4)-glycine hinge mutant (G312A) to 2-Aminoethoxydiphenyl borate (2-APB), an activator of TREK-2, we show that the transduction initiated from pCt domain is allosterically coupled with the conformation of selectivity filter (SF) via the movements of M4, without depending on the original status of SF. Moreover, ΔpCt and G312A also exhibited blunted responses to extracellular alkalization, a model to induce SF conformational transition. These results suggest that the coupling between pCt domain and SF is bidirectional, and M4 movements are involved in both processes. Further mechanistic exploration reveals that the function of Phe316, a residue close to the C-terminus of M4, is associated with such communications. However, unlike TREK-2, M4-hinge of TREK-1 only controls the transmission from pCt to SF, rather than SF conformational changes triggered by pH_o_ changes. Together, our findings uncover the unique gating properties of TREK-2, and elucidate the mechanisms for how the extracellular and intracellular stimuli harness the pore gating allosterically.

Two-pore domain potassium (K2P) channels are expressed in a diverse variety of excitable and non-excitable cells, in which they generate ‘leak’ or ‘background’ currents to stabilize resting membrane potential^1^. TREK-2 (TWIK related potassium channel 2, K2P10.1), a member of TREK/TRAAK (TWIK-related arachidonic acid-stimulated K^+^ channel) K2P subfamily, has been found to play crucial roles in thermosensation, pain perception and pain pathology^2^,^3^.

As a thermo- and mechano-gated K^+^ channel, TREK-2 is modulated by a large volume of stimuli from either extracellular or intracellular side, such as temperature, lysophospholipids, polyunsaturated fatty acids, G-protein coupled receptors, extra- or intracellular pH, and mechanical force^4^,^5^,^6^,^7^,^8^,^9^. To integrate and convert these signals effectively, K2P channels need to open or close its pore actively to control K^+^ efflux, a process termed as gating. Crystal structure studies have suggested that two gates are located oppositely along the ion pathway (pore region) in K2P channels: a selectivity filter gate (SF gate, also called C-type gate, outer gate or inactivation gate, we termed it as SF in this study for simplicity) at the outer end and an inner gate (also called activation gate or bundle-crossing gate) at the intracellular entrance^10^,^11^,^12^. A single K2P molecule is comprised of four transmembrane segments (M1 ~ M4) and two pore-forming domains (P-domain). The inner gate is comprised of the lower parts of inner helix (M2 and M4), and the P-domains form the SF. Despite that progress has been made in identifying input modalities and crystal structures in K2Ps, how these signals control the structure to exert their biological functions remains largely unknown.

Accumulating evidence shows that the fluctuation of extracellular pH (pH_o_) exerts its modulation by inducing the conformational transition of SF in K2P channels^13^,^14^,^15^,^16^,^17^,^18^, including TREK-2^9^,^19^. Most recently, we identified that 2-Aminoethoxydiphenyl borate (2-APB) stimulates the activity of TREK-2 by acting at its cytosolic proximal C-terminus (pCt)^19^. Here, we used pH_o_ and 2-APB as extracellular stimuli and intracellular stimuli, respectively, and combined mutagenesis, recording of K^+^ efflux by two electrode voltage clamp to characterize the gating properties of TREK-2. Our results demonstrate that the pCt domain modulates the long-distance, allosteric coupling to SF via the glycine hinge- and Phe316-induced M4 movements. Moreover, the ability of 2-APB to open the pore does not depend on the original state of SF. In addition, we found that the function of M4-hinge is only associated with 2-APB response of TREK-1, rather than its pH_o_ response, suggesting the facilitation of M4 movement to the conformation change of SF induced by pH_o_ is specific in TREK-2, at least in TREK channels.

## Results

### The proximal C-terminus (pCt) facilitates pore opening without depending the conformation of selectivity filter

**Figure 1:**
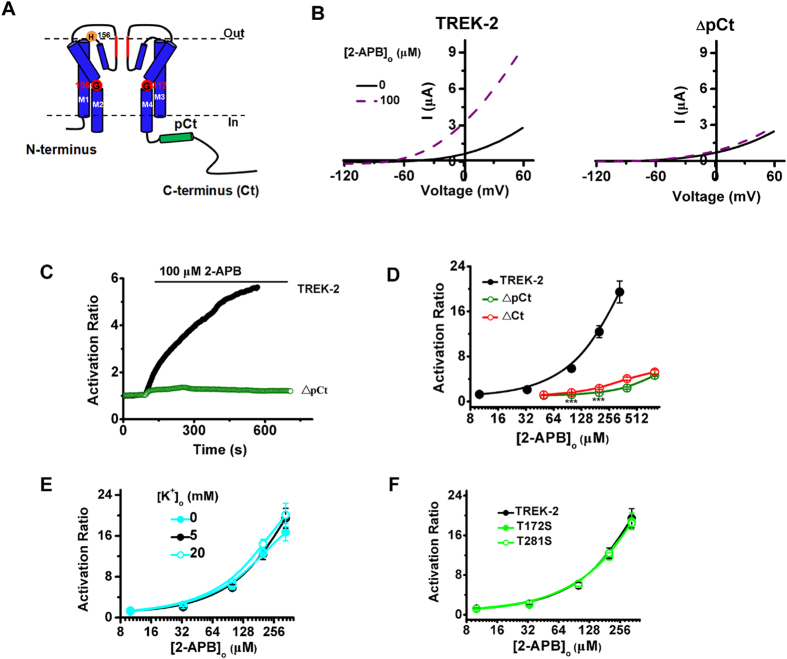
The cytosolic proximal C-terminus controls the pore opening induced by 2-APB without depending the conformation of selectivity filter. (**A**) Cartoon diagram of a single TREK-2 subunit, transmembrane segments 1 ~ 4 (M1 ~ M4), intracellular N-terminus, C-terminus (Ct), proximal C-terminus (pCt) and key residues are indicated. (**B**) Exemplar current-voltage recordings from oocytes expressing TREK-2 or ΔpCt in the presence of 100 μM 2-APB. (**C**) Exemplar current-time recordings from oocytes expressing the WT TREK-2 channels and ΔpCt deletants in the presence of 100 μM 2-APB. (**D**) Comparative analysis of the dose-response curves evoked by 2-APB between TREK-2 and ΔpCt. Due to the lower sensitivity of ΔpCt to 2-APB, higher concentrations were used in the curve. Only two points (100 μM and 200 μM) were tested for statistical significant analysis. (**E,F**) Comparative analysis of the concentration-response curves of TREK-2 channels in the absence and presence of 5 mM, 20 mM extracellular K^+^ (**E**), and the indicated S4 (K^+^ binding site 4) mutants (**F**) activated by 2-APB.

2-APB has been found to activate TREK-2 dramatically and reversibly by primarily acting on the cytosolic pCt domain (Fig. 1A)^19^,^20^, which implicates that this domain potentially facilitates the pore opening. To confirm this, a deletant lack of the pCt fragment (from W326 to A374, ΔpCt) was constructed and expressed in *xenopus* oocytes. As the representative current-voltage (I-V) relationships shown in Fig. 1B, 100 μM 2-APB stimulated the acivity of Wild type (WT) TREK-2 drastically, whereas the ΔpCt channels were only slightly activated. At the measurements of 0 mV, the time course of 2-APB stimulation on TREK-2 (current-time relationships, I-T curve) of representative oocytes at the presence of 100 μM 2-APB was plotted in Fig. 1C. As the time elapsed, the currents of WT channel were enhanced gradually by 2-APB, whereas the currents of ΔpCt channels were elevated a little. The dose-response curves of the two channels were compared in Fig. 1D. The maximal activation ratio (AR_max_, produced by 333 μM 2-APB, unless otherwise specified) was decreased from 19.46 ± 1.94 in WT TREK-2 to 4.64 ± 0.17 in ΔpCt (produced by 800 μM 2-APB, Table 1). Similar with ΔpCt, the full Ct deletant (ΔCt) also exhibited similar 2-APB sensitivity with ΔpCt. These data confirmed the role of pCt in regulating the pore opening induced by 2-APB.

During the pore opening evoked by 2-APB, the selectivity filter (SF) must turn to be conductive, otherwise the accelerated K^+^ efflux could not be achieved. Thus, we speculated that 2-APB is able to open the pore of TREK-2 regardless of the original conformation of SF. Extracellular K^+^ is known to regulate the conformation of SF via a mechanism resembled with C-type inactivation^21^, we then measured the effects of 2-APB on TREK-2 in different concentration of extracellular K^+^ ([K^+^]_o_). As the dose-response curves shown in Fig. 1E, no significant changes in 2-APB response were observed in the absence or presence of 5 and 20 mM [K^+^]_o_ (Table 1).

TREK-1 is the closest relative of TREK-2 in K2Ps^7^. Ba^2+^ inhibits both channels by competitive binding at the putative K^+^ binding site 4 (S4), the innermost location within SF^14^,^22^. Moreover, S4 has been found to be associated with the response of TREK-1 to intracellular acidification^23^. Considering that intracellular acidification also stimulates the activity of TREK-2 and high homology between the two channels^8^, we investigated the responses of the two S4 mutants (T172S and T281S) to 2-APB to assess whether S4 is involved the function of pCt in TREK-2. Different from the effects of intracellular acidification on TREK-1, both T172S and T281S exhibited a similar response upon the application of 2-APB when compared with WT channels (Fig. 1F and Table 1). Taken together, these data reveal that the regulation of pCt domain to pore opening of TREK-2 does not depend on the status of SF.

### The Glycine hinge of M4 (G312) involves the allosteric regulation of pCt domain induced by 2-APB

**Figure 2:**
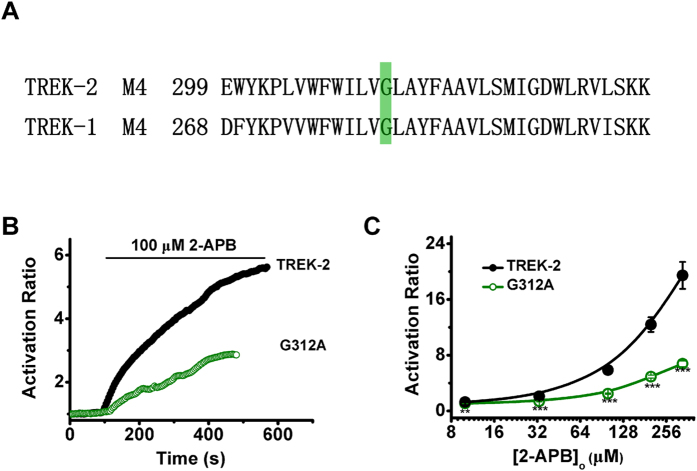
Functional characterization of the Gly312 hinge in the 2-APB response of TREK-2 channels. (**A**) Sequence alignment of the M4 region of indicated channels. The locations of Glycine hinge are indicated. (**B**) Exemplar current-time recordings from oocytes expressing the WT TREK-2 channels and G312A in the presence of 100  μM 2-APB. (**C**) Comparative analysis of the dose-response curves evoked by 2-APB between TREK-2 and G312A.

The cytosolic Ct is covalently linked with M4 in K2P channels, and a Glycine residue located in the middle of M4 serves as a gating hinge that permits the C-terminal parts to bend or straighten to control the inner gate^10^,^12^,^24^. The corresponding residue, G312, is strictly conserved in TREK-2 (Figs 1A and 2A). To understand the role of the position in TREK-2 gating, we investigated the consequence of inhibiting these residues to be kinked in the context of inner helix by replacing them with Ala (G312A). The sensitivity of G312A to 2-APB was measured to evaluate the movements of M4 in the transduction from pCt to SF. As shown in Fig. 2B, the mutation radically reduced the ability of 2-APB to activate TREK-2, and the AR_max_ was decreased to 6.76 ± 0.43 (Fig. 2C and Table 1). Obviously, the allosteric coupling from pCt to SF induced by 2-APB facilitated by M4 movements in TREK-2.

### SF conformational transition is also coupled with proximal Ct domain

**Figure 3:**
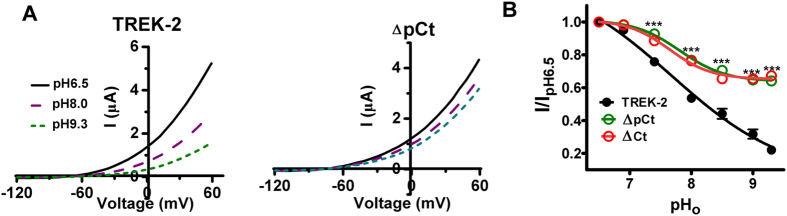
The cytosolic proximal C-terminus is involved in the gating process of selectivity filter induced by extracellular alkalization. (**A**) Exemplar current-voltage recordings from oocytes expressing TREK-2 or ΔpCt as pH_o_ transitions among 6.5, 8.0 and 9.3. (**B**) Normalized responses of indicated channels to pH_o_ changes. The currents were normalized by the ones recorded at pH6.5 (I_pH6.5_).

As mentioned above, intracellular stimuli acting at pCt is able to open the pore via the motions of M4. Nevertheless, whether extracellular signal utilizes similar pathway remains to be explored. External alkalization inhibits TREK-2 currents by inducing SF collapse to narrow the pore after sensed by His156 (Fig. 1A)^9^,^19^. Thus, extracellular pH (pH_o_) gating kinetics is a ready model to measure such conformational change in SF^9^,^14^,^17^,^25^. To investigate the possible role of the pCt domain in SF gating, we examined the response of ΔpCt to enhanced pH_o_. Despite that the inhibitory effects were clearly observed in WT channels when pH_o_ was enhanced from 6.5 to 8.0 or 9.3, such effects on ΔpCt were severely impaired (Fig. 3A). Accordingly, the dose-response curve of the deletant exhibited a slowdown and blunted tendency. The minimal normalized current of external alkalization (I_pH9.3_/I_pH6.5_) on WT TREK-2 was 0.22 ± 0.02, whereas the factor for ΔpCt was 0.64 ± 0.02 (Fig. 3B and Table 1). Consistent with the results from ΔpCt, ΔCt displayed similar sensitivity with ΔpCt to pH_o_ (Fig. 3B). These results demonstrate the cytosolic pCt domain serves as not only a 2-APB acting site, but also a gating element controlling the movements of SF.

### M4-Glycine hinge (G312) also controls the transduction from selectivity filter to proximal C-terminus

**Figure 4:**
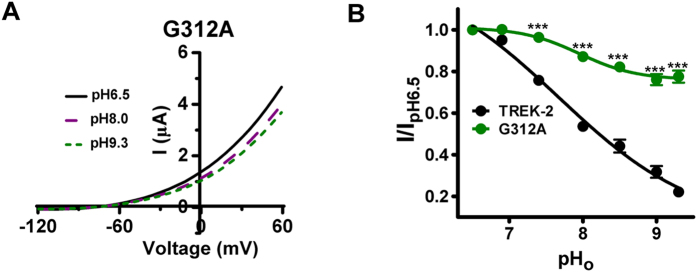
Functional characterization of the Gly312 hinge in the pHo response of TREK-2 channels. (**A**) Exemplar current-voltage recordings from oocytes expressing the WT TREK-2 channels and G312A as pH_o_ transitions among 6.5, 8.0 and 9.3. (**B**) Concentration-dependence for extracellular alkalization inhibition of TREK-2 and G312A.

Considering that the movement of SF evoked by pH_o_ changes is facilitated by the pCt domain in TREK-2, we proposed that G312 might also be involved in such long-distance transduction. Test of G312A to extracellular alkalization revealed that the mutant indeed displayed blunted response (Fig. 4A), with an increased I_pH9.3_/I_pH6.5_ of 0.78 ± 0.03 (Fig. 4B and Table 1). Therefore, these data indicate that motion of M4 is essential in regulating the transduction from extracellular sensor to intracellular pCt domain.

### Phe 316 (F316) is involved in the bidirectional coupling between SF and pCt domain

**Figure 5:**
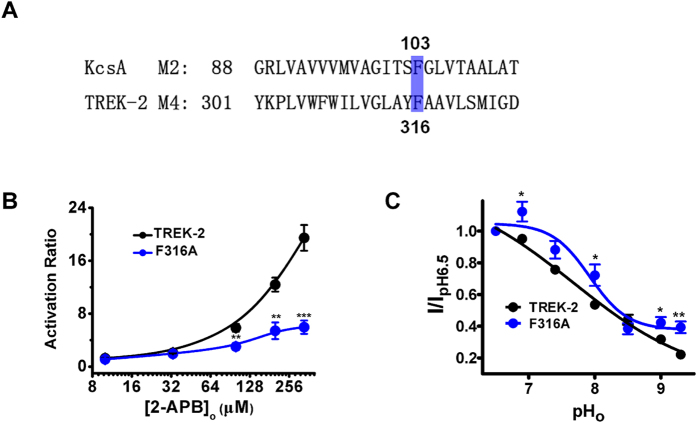
Functional characterization of the Phe316 in the 2-APB and pH_o_ response of TREK-2 channels. (**A**) Sequence alignment between TREK-2 M4 and KcsA M2. The location of Phe316 is indicated. (**B**) Comparative analysis of the dose-response curves evoked by 2-APB between TREK-2 and G312A. (**C**) Normalized responses of indicated channels to pH_o_ changes.

Structural analysis of KcsA channel (Potassium channels from *S. lividans*) reveals that Phe 103, a residue close to the C-terminal end of the M2 helix, changes its rotameric conformation as a consequence of the hinge-bending and rotation of M2 to destabilize its SF^26^,^27^. This residue is strictly conserved in M4 of TREK-2 (F316, Fig. 5A). Small side chain substitutions at position 103 severely impair the allosteric coupling between inner gate and SF in KcsA^27^, we thus also mutated F316 of TREK-2 to Alanine (F316A) to investigate its function in the bidirectional couplings. Evaluation of the mutant to 2-APB activation demonstrated that the transition led to less sensitive response compared with WT channels, with a decreased AR_max_ of 5.96 ± 1.02 (Fig. 5B). Likewise, F316A also exhibited slightly less sensitive to extracellular alkalization compared with WT channels (I_pH9.3_/I_pH6.5_ = 0.39 ± 0.04, Fig. 5C). These data provide strong functional evidence demonstrating that the bidirectional coupling between SF and inner gate is facilitated by the function of F316 in TREK-2 channels.

### The facilitation of M4 movements to SF gating is specific for TREK-2 in TREK subfamily

**Figure 6:**
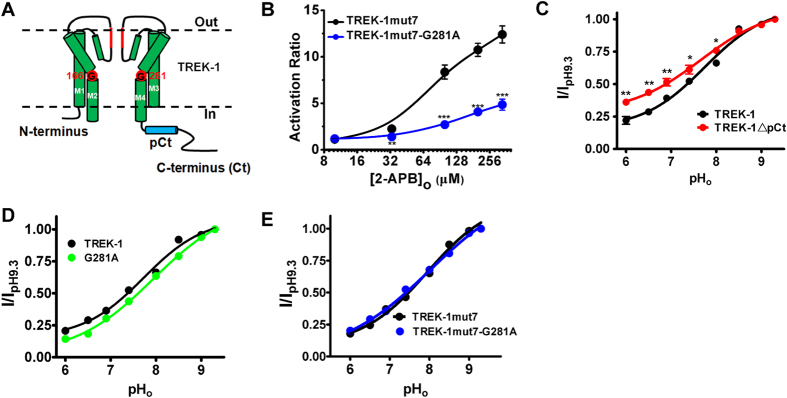
Delineation the roles of Glycine hinges in TREK-1 channels. (**A**) Cartoon diagram of a single TREK-1 subunit, transmembrane segments 1 ~ 4 (M1 ~ M4), the intracellular N-terminus, C-terminus (Ct), proximal C-terminus (pCt) and the key residues are indicated. (**B**) Quantification of the effect of 2-APB on the indicated channels. (**C–E**) Comparative analysis of pH_o_ response curves for the indicated channels.

The next question we asked was whether the above mechanism of gating transduction was common to K2Ps or unique to TREK-2. Considering that TREK-1 is the nearest relative of TREK-2 in K2P family, we characterized its gating mechanism by investigating the behavior (2-APB or pH_o_ sensitivity) of Gly hinge mutants (G281A for M4) and pCt deletant (from I292 to C365, TREK-1ΔpCt) (Fig. 6A).

TREK-1 is insensitive to 2-APB^20^, but mutation of the seven residues within its pCt domain to the equivalent ones of TREK-2 (TREK-1mut7) were found to recover its sensitivity^19^. Thus, this mutant was used to investigate whether M4 movements participated in the long-distance transduction from pCt to SF in TREK-1. We put G281A substitution in the context of TREK-1mut7 to construct TREK-1mut7-G281A. Measurement of its sensitivity to 2-APB indicated that such substitution caused a marked reduction in the ability of the compound to activate TREK-1mut7, and the AR_max_ was decreased from 12.40 ± 0.92 in TREK-1mut7 to 4.85 ± 0.60 in TREK-1mut7-G281A (Fig. 6B and Table 1). Obviously, similar with TREK-2, the pCt-regulated pore opening in TREK-1 is also controlled by the movements of M4.

TREK-1 was inhibited by acidic pH_o_ via a sensor, His126^17^. When pH_o_ was enhanced gradually, TREK-1ΔpCt showed a decreased sensitivity compared with WT TREK-1 (Fig. 6C and Table 1), suggesting that the transduction from His126 is also facilitated by intracellular pCt domain. To investigate whether the movements of M4 involves this allosteric coupling evoked by extracellular signal, pH_o_ sensitivity of M4 mutants, G281A and TREK-1mut7-G281A, was assessed. Different from TREK-2, no significant changes in pH_o_-induced gating kinetics were observed between WT and G281A (Fig. 6D and Table 1), or TREK-1mut7-G281A (Fig. 6E and Table 1). These data indicate that the M4 movement of TREK-1 is only involved in the transduction evoked by intracellular stimuli, instead of the one by extracellular stimuli. Thus, the facilitation of M4 movements to SF gating is specific for TREK-2 in TREK subfamily.

## Discussion

Although the function and importance of TREK-2 channel have recently been deciphered and recognized^2^,^3^, the molecular mechanism of this channel gating remains to be elucidated. In the present study, we characterized the gating kinetics of TREK-2 channels in response to 2-APB and pH_o_ changes, which represents intracellular stimuli and extracellular stimuli, respectively. Our results identified the mechanism regulating the allosteric coupling between SF and cytosolic C-terminus in TREK-2, and also the difference between TREK-2 and TREK-1 in gating properties.

Crystal structure of several K2Ps have been solved in recent years^10^,^11^,^12^,^28^,^29^, but only a short segment of Ct was included in these structures. Thus, the function of the Ct, especially the pCt domain, in the context of the whole channel has not been identified. Our data showed that the stimulatory ability of 2-APB, an intracellular stimuli to TREK-2, was radically decreased by deletion of pCt or Ct fragment. In addition, opening of the pore of TREK-2 by 2-APB is affected by neither the conformational alteration of SF gate induced by fluctuation of [K^+^]_o_ nor mutations of the S4 site (Fig. 1). These results clearly suggest that the cytosolic pCt domain facilitates the pore opening without depending on the conformation of SF, implicating the allosteric coupling from pCt domain to SF.

Structurally, SF is located at the outer of pore, and the pCt resides at the intracellular side of the channel (Fig. 1A). Thus, they are separated by a relatively long distance. Considering that M4 of K2Ps serves as pore-lining helix and is covalently linked with Ct, we reasonably presumed that the movement of M4 might be involved in the long-distance coupling from pCt to SF. Indeed, we found that substitution of the putative Glycine hinge (Gly312), whose flexibility might allow the post-Gly hinge segments of M4 to bend or twist to fulfill regulations by stimuli^12^,^28^, rendered TREK-2 less sensitive to 2-APB stimulation (Fig. 2). The Glycine corresponds to the ones that are central to the gating of diverse potassium channels^27^,^30^,^31^,^32^,^33^,^34^,^35^. Recently, consensus has been emerged that Ct might exert its roles by manipulating the inner gate, as illustrated in inwardly rectifying potassium (Kir) channels^36^,^37^,^38^,^39^, cyclic nucleotide-regulated ion channels^40^ and tetrameric sodium channels^41^. To respond to stimuli, the Ct domains undergo a rotational movements relative to the plane of the membrane, and then translating the twist configuration into opening the inner gate in Kir channels^31^,^42^,^43^. Moreover, through modulating the movement of inner gate, the pCt domain might eventually control the extent of conformational change at SF gate, as suggested in KcsA channels^44^. These studies provide the possible mechanisms for how the Ct domain modulates the SF conformation, in which the movement of inner gate involves in such long-distance, allosteric transduction. Our results suggest that TREK-2 might also use similar mechanisms. The movement of M4 induced by its glycine hinge is necessary for the transduction from pCt domain to SF, whereas the inner gate of K2Ps are composed of the post-hinge of M2 and M4. Thus, despite that whether the inner gate works as a gate is still in paradox in K2Ps^15^,^23^,^32^,^33^,^45^, our results support that the inner gate indeed functions as a channel gate.

Except for M4, M2 also serves as pore-lining helix. Crystal structures of TRAAK^28^,^29^ and TREK-2^12^ suggest that the movements of M4 is facilitated by the substantial buckling of M2. Our previous study suggests that the bottom of M2 is the putative secondary binding site of 2-APB to TREK-2^19^, further supporting the crucial roles of M2 in pore gating. According to the sequence of TREK-2, G196 in M2 (Fig. 1A) is the corresponding hinge of TREK-2-M4 and inner helix of most classic potassium channels. In K2P channels, data from TASK3^33^ and chimera of KCNK0/*shaker*^32^ have verified such hinge function. In addition, a “GXG” downstream the G196 (especially in the TREK/TRAAK subclass) also exerts hinge function during gating^12^,^28^, implying the more complex properties of M2 in K2P channels.

The activity of TREK-2 is regulated not only by intracellular stimuli, but also by the signals from outside of the membrane. Among them, the sensitivity of the channel to pH_o_ changes is the most studied model with a clear mechanism, in which alkalized pH_o_ induces the nonconductive conformation of SF to narrow the pore, and ultimately blocks the ion efflux^9^. However, is the nonconductive SF sufficient to close the pore? Does any other gating element cooperatively facilitate the process? To address these questions, we measured the pH_o_ responses of ΔpCt and G312A. Intriguingly, both ΔpCt and G312A exhibited decreased pH_o_ sensitivity compared with WT TREK-2 (Figs 3 and 4), demonstrating that the pCt domain and the movement of M4 are also required to achieve the conformational transition of SF. These critical observations also suggest that the allosteric coupling in TREK-2 between pCt domain and SF is bidirectional, in which its pCt domain and M4 glycine hinge serve as common gating elements.

To further strengthen the above-mentioned notion, the possible mechanism underlying the coupling was also explored. Transition of F316 (a residue downstream of G312 and close to the C-terminus of M4) to Ala blunted the effects of 2-APB and pH_o_ on TREK-2 (Fig. 5), suggesting the function of F316 is involved in the bidirectional coupling. Consistent with previous studies^26^,^27^, our data imply that the allosteric coupling between inner helix and SF might rely on straightforward steric contacts in K^+^ channels, since reduction of the side chain volume of F316 limits the communications between M4 (or pCt domain) and SF. Thus, although they are distinct in structure, K2Ps might share similar gating mechanism that controlling the communications between gates. Notably, compared with ΔpCt and G312A, F316A exhibits different attributes: although all the three mutations destroy the 2-APB effects on TREK-2 (Figs 1D, 2C and 5B), and ΔpCt and G312A also becomes less sensitive to pH_o_ (Figs 3B and 4B), the ability of alkalized pH_o_ to inhibit TREK-2 only slightly decreased by F316 to A transition (Fig. 5C). Accordingly, we speculate that F316 mainly participate in the coupling from pCt domain to SF (outward), rather that the direction from SF to pCt. However, more data are still required to support the idea.

To identify that whether the gating mechanism of TREK-2 is common in K2Ps, we also characterized the gating properties of TREK-1. Similar with TREK-2, 2-APB is able to open the pore of TREK-1 if the acting site of the compound is transplanted into the pCt domain^19^, and pCt deletant exhibits blunted sensitivity to pH_o_ changes (Fig. 6C). These results strongly suggest that the pCt domain also controls the bidirectional transduction induced by 2-APB and pH_o_ changes. In addition, M4-hinge modulates the stimulatory effects of 2-APB on TREK-1mut7 (Fig. 6B), supporting the idea that the movements of M4 also induces the allosteric regulation from pCt to SF in TREK-1. However, the M4-hinge appears only function in the pH_o_ changes-induced transduction in TREK-2, instead of in TREK-1. Because neither of G281A and TREK-1mut7-G281A, the M4-hinge mutants of TREK-1 exhibited blunted response to pH_o_ changes (Fig. 6D,F). Similar phenomenon was also found in TASK3 (K2P9.1), which demonstrate that the Glycine hinge of inner helix is not involved in the regulating status of SF gate^33^. Obviously, different mechanism from that of TREK-2 might modulate the allosteric coupling from SF to pCt in TREK-1. Although the same set of gating apparatus is utilized by K2Ps (they have similar structures), the gating mechanism of TREK-2 appears to be unique. The reason for such difference is still an open issue, and future structural data of TREK-1 or TASK3 might help to explain.

In conclusion, we have established that the pCt domain controls the allosteric regulations evoked by pH_o_ changes and 2-APB via inducing the movement of M4 in TREK-2 channel by using mutagenesis and two electrodes voltage clamp. In our proposed model, binding of 2-APB might result in the conformational change of the cytosolic pCt domain, which tranduces the signal to M4 and lead to its motions around its glycine hinge and F316, and ultimately forces the SF to be conductive, regardless of its original status. Conversely, extracellular alkalization induces the collapse of SF, and then propagates the signal to the pCt domain through the motions of M4, which might finally lead to the closure of inner gate. Unlike TREK-2, despite that the motions of M4 also controls the allosteric transduction initiated from pCt domain in TREK-1, they are not involved in the transduction from extracellular sensor to intracellular pCt domain. Taken together, we identified a unique gating mechanism for how extra- and intracellular stimuli control the pore in TREK-2.

## Methods

### Molecular Biology

The human TREK-2 (NM_138318)- and TREK-1 (NM_014217) -expressing vector (pGH19-TREK-2) was constructed as described^19^. On the basis of this vector, point Mutations and deletions were engineered using the MutanBEST kit (TaKaRa, Dalian, China)-guided high-fidelity PCR. All the mutations were confirmed by DNA sequencing. All the Plasmids were linearized by *X*ho I before in vitro transcription. cRNA was synthesized using the RiboMAX™ Large Scale RNA Production Systems (Promega, Madison, WI) kit.

### Channel expression in *Xenopus* oocytes

Pieces of ovarian lobes were excised from *Xenopus laevis*. Stage V or VI oocytes were separated by collagenase (Sigma Aldrich, St Louis, MO) digestion. 0.1 ~ 10 ng cRNA (46 nl in volume) was microinjected into each oocyte. Injected cells were incubated at 18 °C in ND96 medium. All experimental procedures were approved by the Ethical Committee for Animal Experimentation of Beijing Institute of Pharmacology and Toxicology, and in accordance to the regulations of the Institutional Review Committee on Animal Care and Use.

### Electrophysiology

For TEVC measurement, microelectrodes were pulled with a tip resistance of 0.1–1 MΩ and were filled with 3 M KCl. Recordings were performed under constant perfusion at room temperature, and in standard, physiological extracellular solution (standard solution) unless otherwise indicated. Whole-cell currents were measured 1–3 days after injection, and amplified using an Axoclamp2B amplifier (Axon Instruments, Union City, CA) in TEVC mode. Data were sampled at 2 kHz and filtered at 0.5 kHz with Clampex 10.0 software (Axon Instruments). K^+^ currents were elicited by continuous voltage-ramps from −120 to +60 mV from a holding potential of −80 mV, with 2 s in duration (current-voltage relationship, I–V curve). The currents of TREK-2 undergo obvious “run-up” at the beginning of recording, thus, the current of each measurement was stabilized for about 20 min before applying stimulus. 2-APB was applied externally. For each concentration of 2-APB and specified pH_o_, the recorded currents through all the channels used were under the condition of continuing perfusion, until the steady state of 2-APB or pH_o_ effect was achieved.

### Data analysis

All the ratios were calculated using the currents recorded at 0 mV. In TREK-1 and TREK-2, Activation ration (AR) was calculated from I_2-APB_/I_o_, where I_2-APB_ represented the currents in the presence of 2-APB, and I_o_ was baseline currents in the absence of 2-APB. I/I_pH6.5_ was used to calculate pH_o_ effects, where I represented the currents in a specific pH, and I_pH6.5_ was the currents recorded at pH6.5. Because TREK-1 is activated by alkalized pH_o_, I/I_pH9.3_ was calculated as the function of different extracelluar pH_o_. Data were analyzed with origin 8.0 (OriginLab Corporation, Northampton, MA) and GraphPad prism version 5.0 (GraphPad Software Inc., La Jolla, CA). Concentration-response curves were fitted to the Hill equation. All statistical values were presented as mean ± SEM for the number of measurements indicated (n ≥ 5 from at least two batches of oocytes). Statistical significance was determined by using unpaired Student’s t test and indicated as follows: *P < 0.05; **P < 0.01; ***P < 0.001. The AR_max_, I_min_I_max_ and the number of oocyte used in each point of these curves were listed in Table 1.

### Chemicals and solutions

Standard solution (5 mM K^+^) (5 mM KCl, 93 mM NaCl, 1 mM MgCl_2_, 1.8 mM CaCl_2_, 5 mM HEPES, pH7.4, adjusted with NaOH), 0 mM K^+^ (98 mM NaCl, 1 mM MgCl_2_, 1.8 mM CaCl_2_, 5 mM HEPES, pH7.4, adjusted with NaOH), 20 mM K^+^ (20 mM KCl, 78 mM NaCl, 1 mM MgCl_2_, 1.8 mM CaCl_2_, 5 mM HEPES, pH7.4, adjusted with NaOH), ND96 (96 mM NaCl, 2 mM KCl, 1.8 mM CaCl_2_, 1 mM MgCl_2_, 10 mM HEPES, 5 mM pyruvate, 100 mg/ml gentamycin, pH 7.2). 2-APB (Promega, Madison, WI) was diluted with the standard solution freshly.

## Additional Information

**How to cite this article**: Zhuo, R.-G. *et al.* Allosteric coupling between proximal C-terminus and selectivity filter is facilitated by the movement of transmembrane segment 4 in TREK-2 channel. *Sci. Rep.* **6**, 21248; doi: 10.1038/srep21248 (2016).

## Figures and Tables

**Table 1:** 2-APB and pH_o_ response of indicated channels.

Channel	*2-APB response*		*pH*_*o*_*response*	
	AR_max_^a^	n^b^	I_min_/I_max_^c^	n^b^
TREK-2, 5 mM [K^+^]_o_	19.46 ± 1.94	7–19	0.22 ± 0.02	13–15
ΔpCt	4.64 ± 0.17	5	0.64 ± 0.02	7
G312A	6.76 ± 0.43	5–6	0.78 ± 0.03	8–9
TREK-2, 0 mM [K^+^]_o_	16.72 ± 1.72	5–11	–	
TREK-2, 20 mM [K^+^]_o_	20.38 ± 1.30	7–12	–	
T172S	18.84 ± 1.65	5–11	–	
T281S	18.44 ± 1.19	7–11	–	
F316A	5.96 ± 1.02	6–7	0.39 ± 0.04	7–8
TREK-1	–		0.20 ± 0.03	7–8
TREK-1ΔpCt	–		0.36 ± 0.02	7
G281A	–		0.14 ± 0.02	6–7
TREK-1mut7	12.40 ± 0.92	5–7	0.18 ± 0.03	6
TREK-1mut7-G281A	4.85 ± 0.60	5–7	0.20 ± 0.01	6

^a^The maximal concentrations of 2-APB used in these curves is 333 μM for these channels, except for ΔpCt (800 μM).

^b^The number of oocytes used for each point in concentration response curves.

^c^For TREK-2, I_min_/I_max_ means I_pH9.3_/I_pH6.5_; For TREK-1, I_min_/I_max_ means I_pH6.0_/I_pH9.3_. –Not determined.
